# Retinoic Acid as a Modulator of T Cell Immunity

**DOI:** 10.3390/nu8060349

**Published:** 2016-06-13

**Authors:** Maria Rosa Bono, Gabriela Tejon, Felipe Flores-Santibañez, Dominique Fernandez, Mario Rosemblatt, Daniela Sauma

**Affiliations:** 1 Departamento de Biologia, Facultad de Ciencias, Universidad de Chile, Las Palmeras 3425, Ñuñoa, Santiago 7800003, Chile; mrbono@uchile.cl (M.R.B.); Gabrielatejon@gmail.com (G.T.); felipeflores.uchile@gmail.com (F.F.-S.); dominique.fernandez.q@gmail.com (D.F.); mrosemblatt@cienciavida.org (M.R.); 2 Fundacion Ciencia & Vida, Santiago 7780272, Chile; 3 Facultad de Ciencias Biologicas, Universidad Andres Bello, Santiago 8370146, Chile

**Keywords:** retinoic acid, vitamin A, T cell, oral tolerance, homing

## Abstract

Vitamin A, a generic designation for an array of organic molecules that includes retinal, retinol and retinoic acid, is an essential nutrient needed in a wide array of aspects including the proper functioning of the visual system, maintenance of cell function and differentiation, epithelial surface integrity, erythrocyte production, reproduction, and normal immune function. Vitamin A deficiency is one of the most common micronutrient deficiencies worldwide and is associated with defects in adaptive immunity. Reports from epidemiological studies, clinical trials and experimental studies have clearly demonstrated that vitamin A plays a central role in immunity and that its deficiency is the cause of broad immune alterations including decreased humoral and cellular responses, inadequate immune regulation, weak response to vaccines and poor lymphoid organ development. In this review, we will examine the role of vitamin A in immunity and focus on several aspects of T cell biology such as T helper cell differentiation, function and homing, as well as lymphoid organ development. Further, we will provide an overview of the effects of vitamin A deficiency in the adaptive immune responses and how retinoic acid, through its effect on T cells can fine-tune the balance between tolerance and immunity.

## 1. Introduction

In the early 20th century, several researchers performed studies on laboratory animals to identify essential dietary components. Hopkins, McCullum, Osborne, and Mendel reported that animals fed with only fats, proteins, starch and inorganic salts, were more susceptible to infections and failed to grow normally. Administration of the factors present in dairy products and cod liver oil prevented these complications. McCollum called this factor “fat-soluble accessory” [1]. In 1928, Green and Mellanby reported that vitamin A, played an important role in conferring resistance to many infections and declared vitamin A as an anti-infective agent [2]. The role of vitamin A in immunological fitness was confirmed in randomized clinical trials performed in the 1980–1990s studying the effect of vitamin A supplementation on child mortality in Africa and Asia. These trials resulted in a significant reduction of mortality among children of 1–5 years of age where most of the deaths prevented were associated with less severe clinical manifestations of measles and diarrheal disease [1,3]. Furthermore, although several studies have reported that vitamin A increases antibody production which has led to the supplementation of vaccines with vitamin A, results from human immunization programs have not shown a significant effect on vaccine efficacy upon vitamin A supplementation [4]. Despite all these efforts, today vitamin A deficiency (VAD) is still one of the most common micronutrient deficiencies, affecting approximately one-third of the world’s preschool-age population [5] and remains the primary cause of immunosuppression worldwide [6]. In recent years, and with the development of better models and experimental techniques, it has become clear that vitamin A, through its metabolite retinoic acid (RA), has a broad impact on diverse aspects of the immune response, including mucosal immunity, tolerance, leukocyte trafficking and lymph node organogenesis.

## 2. Vitamin A Metabolism and Retinoic Acid Signaling

Dietary vitamin A is obtained through the consumption of foods containing vitamin A precursors, such as carotenoids, or vitamin A in the form of retinyl esters. The proximal portion of the gut is the primary tissue where vitamin A precursors, like β-carotene, are converted to retinoids in the lumen by enterocytes. Dietary precursors are incorporated into nascent chylomicrons and enter the general circulation. Following their absorption and arrival in the circulation, retinyl esters are stored in the liver, where they are hydrolyzed into retinol and delivered into the circulation [7,8].

The three active forms of vitamin A (retinal, retinol and retinoic acid) serve different physiological functions. For instance, 11-cis retinal plays an important role in vision as it functions as a light-sensitive chromophore that binds and maintains the stability of rhodopsin [9]. On the other hand, retinol circulates in plasma, bound to the Retinol Binding Protein (RBP), and in this form serves as a source of RA [10]. RA, the main vitamin A metabolite, has been involved in the control of gene expression in a variety of processes including immune function.

RA is generated from retinol after two sequential reactions. The first reaction involves the reversible conversion of retinol to retinaldehyde, also known as retinal. Initially, it was reported that this reaction was catalyzed by a subfamily of alcohol dehydrogenases (ADHs) that include ADH1, ADH5 and ADH7 [11,12]. However, more recent evidence has demonstrated that these ADHs do not contribute to RA synthesis under normal physiological conditions and that only short-chain dehydrogenases/reductases such as microsomal retinol dehydrogenases (RDHs) are the physiologically relevant enzymes in the conversion of retinol to retinaldehyde [13]. In a second reaction, retinal is irreversibly converted to RA by one of the three retinal dehydrogenase isoforms RALDH1, RALD2 and RALDH3, which form a subfamily of class I aldehyde dehydrogenases [12]. Since RALDH expression is tightly regulated, this enzyme is considered the key enzyme defining particular cell populations that are able to produce RA [14].

Most of the immunological functions associated with vitamin A are mediated by RA in the form of all-trans-RA and 9-cis-RA. RA binds to nuclear receptors, including retinoic acid receptors (RAR), retinoid X receptors (RXR) and PPARβδ. The RAR family of receptors includes three receptors: RARα, RARβ and RARγ [15]. RARs form heterodimers with RXR to regulate target gene expression through the binding to retinoic acid-responsive elements (RARE) [16]. Both all-trans-RA and 9-cis-RA are ligands of the nuclear receptors RARα, RARβ, and RARγ, whereas only 9-cis-RA can bind nuclear RXR [17,18].

RAR/RXR heterodimers constitutively bind to RAREs. In the absence of ligands, RAR/RXR heterodimers recruit corepressors to inhibit the transcription of target genes, whereas the binding of RAR/RXR ligands displaces the corepressors, allowing the recruitment of coactivators and resulting in the transcriptional activation of target genes [19,20,21]. Most of the RA immune-related functions signal through the RAR/RXR pathway, which is driven by all-trans-RA acting through RARα [22].

## 3. Retinoic Acid and T Cell Homing

Following their activation in lymph nodes, in order to execute their function, antigen-experienced T cells must migrate into different tissues following a multistep cascade of events. The preferential trafficking to a specific tissue, known as T cell “homing”, is tightly regulated by the expression of different adhesion molecules or distinct “molecular zip codes” on T cells [23]. These adhesion molecules allow T cells to bind to addressins present in the endothelium allowing for their arrest and entry to target sites [24].

Adhesion molecules involved in T cell homing to the intestine have been extensively studied. It has been demonstrated that the expression of the α4β7 integrin and CCR9 chemokine receptor by T cells is crucial for T cell migration to the gut in absence of inflammation [23,25]. CCL25, the ligand of CCR9, is expressed by intestinal epithelial cells whereas Mucosal Addressin Cell Adhesion Molecule-1 (MAdCAM-1), the ligand of α4β7 is expressed by high endothelial venules in gut-associated lymphoid tissue [24].

**Figure 1:**
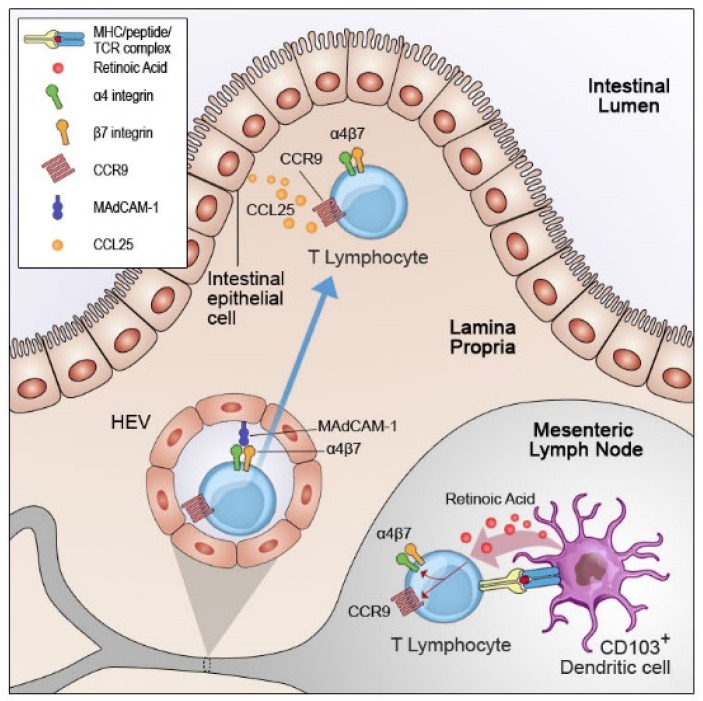
Retinoic acid induces T cell homing to the gut. A specialized subset of gut-resident dendritic cells which express CD103 integrin is able to produce retinoic acid during the interaction with T cells in the mesenteric lymph node. Retinoic acid induces the expression of α4 which binds to β7 forming the α4β7 integrin. α4β7 integrin binds to the mucosal addressin cell adhesion molecule 1 (MadCAM-1) which is expressed by high endothelial venules (HEV) in the intestinal tissue. Retinoic acid also induces the expression of the chemokine receptor CCR9, which binds to CCL25 chemokine produced by intestinal epithelial cells. The expression of α4β7 and CCR9 allows the trafficking of T cells to the lamina propria.

The factors determining the induction of specific adhesion molecules on T cells were discovered few years ago. In 2003, our group reported that dendritic cells (DCs) are able to instruct T cells to migrate to different tissues. In this study, intestinal DCs from Peyer’s patches and mesenteric lymph nodes but not from spleen or peripheral lymph nodes were able to induce the gut-homing receptors α4β7 and CCR9 on CD8^+^ T cells [26]. Shortly after that study, it was demonstrated that RA is the factor responsible in the induction of gut-homing receptors on CD4^+^ T cells [27] (Figure 1), CD8^+^ T cells [28] and IgA-secreting B cells [29,30]. Interestingly, RA not only induces gut-homing receptors on T cells but also inhibits skin homing receptors, such as E-Selectin ligands [27]. Most importantly, CD4^+^ and CD8^+^ T cell homing to the small intestine lamina propria is impaired in VAD mice, confirming the essential role of RA in T cell homing to the gut [27]. The effect of RA on α4β7 and CCR9 expression is mediated by RARα which binds to RAR-response elements within the regulatory region of the gene that encodes α4 [31,32], and forms RARα/RXR heterodimers that bind to a RARE in the promoter region of CCR9 [33].

After the discovery of RA as a master controller of T cell homing to the gut, special focus has been placed on determining the cellular source of RA. DCs from Peyer’s patches and mesenteric lymph nodes express RALDH1 and RALDH2 mRNAs, respectively, which are the key enzymes for the conversion of vitamin A to RA. In contrast, DCs obtained from spleen express only marginal levels of RALDH1 mRNA and essentially no RALDH2 and RALDH3 mRNAs. Furthermore, DCs isolated from Peyer’s patches and mesenteric lymph nodes, but not those isolated from spleen produce RA from retinol when co-cultured with T cells and their specific antigen [27]. Further studies have shown that a subset of DCs that are enriched in the small intestine and express the CD103 integrin, present high levels of RALDH2 mRNA and thus are specialized in the production of RA [34,35,36].

Others have reported that RA may be produced locally at sites of inflammation [37]. This finding raises the intriguing possibility that RA may also be involved in the migration of T cells to inflamed sites. RA is able to induce α4 integrin which, in addition to forming heterodimers with β7, may also form heterodimers with integrin β1. α4β1, in turn, binds to Vascular Cell Adhesion Molecule-1 (VCAM-1), which is upregulated by the endothelium during inflammation [24]. Along these lines, using skin transplantation as a model of inflammation, Pino-Lagos and collaborators have shown that T cells deficient in RA signaling have altered migration to sites of inflammation, such as the skin graft [37].

## 4. Role of Retinoic Acid in Regulatory T Cell Differentiation 

The intestine is colonized by a vast community of commensal microbes and is constantly exposed to dietary antigens. However, the intestinal immune system has strategically evolved to efficiently discriminate between these innocuous stimuli and pathogenic microorganisms. Oral tolerance is defined as a state of local and systemic immune unresponsiveness that is induced by the oral administration of an innocuous antigen. It is now recognized that regulatory T cells (Tregs) are crucial in maintaining immune homeostasis and tolerance to commensal microbiota and food proteins in the gut [38].

Regulatory T cells constitute a subset of CD4^+^ T cells responsible for inducing and maintaining peripheral tolerance [39]. Depending on their origin, Tregs can be classified into two subpopulations: thymus-derived Tregs, which are generated in the thymus and circulate in the periphery as functional mature Tregs [40], and peripherally derived Tregs, which are generated in the periphery from CD4^+^ naive T cells in the presence of cytokines such as TGF-β and IL-2 [41,42,43]. Both populations express the transcription factor Foxp3, the master regulator of Treg cell differentiation and effector functions [44].

Evidence of the role of extrathymic Treg differentiation in the induction of oral tolerance was presented by the Belkaid group in 2007, showing that the small intestine harbors the conditions for the *de novo* generation of Tregs. Transferred naive T cells can be converted to Tregs in the gut-associated lymphoid tissue where gut-associated DCs, through the production of RA, are responsible for this conversion [45]. This effect of RA on Treg differentiation was shown to be mediated through RARα [46,47]. Since that seminal report, several groups have demonstrated that high concentrations of RA in combination with TGF-β induce the expansion of murine [35,45,48,49,50] and human Tregs *in vitro* [32,51,52,53,54].

Retinoic acid also induces Treg conversion indirectly, through the inhibition of cytokine production by a population of memory T cells that blocks the differentiation of naive T cells into Tregs [54,55,56,57]. This population of memory T cells (CD44^hi^) produces IL-4, IL-21 and IFN-γ and inhibits Treg cell differentiation *in vitro*. The addition of RA in culture limits cytokine production by these memory T cells and thus enhances Treg differentiation.

As pointed out above, RA synergizes with TGF-β not only to induce the expansion of Tregs but it has been shown to sustain the stability and function of these cells during inflammation. We and others have shown that murine Tregs generated in the presence of RA present a more stable phenotype than Tregs differentiated without RA , even after these cells are challenged *in vivo* with their cognate antigen [49,50] or in a setting of intestinal inflammation [58]. Zhou and collaborators have shown that Treg stability during collagen-induced arthritis is dependent on the reduction of IL-6 receptor α expression in Tregs generated in the presence of RA [59]. In addition, RA enhances TGF-β signaling by increasing the expression and phosphorylation of Smad3, a transcription factor that regulates the expression of Foxp3. This results in increased Foxp3 expression, even in the presence of Th17-inducing cytokines, such as IL-6 or IL-21 [60,61]. RA in conjunction with TGF-β are also important for the stability of human *de novo* generated Treg and thymus-derived Tregs, demonstrated by the maintenance of Foxp3 expression and suppressive function [52,54]. However, thymus-derived human Tregs maintained with RA alone lose their regulatory properties and differentiate into pro-inflammatory cells during an inflammatory response [51,53].

Several studies have shown that RA not only promotes the differentiation, stability and function of murine and human Tregs but also induce the expression of gut-homing receptors in these cells [49,50,53,62]. Despite this finding, Tregs generated in the presence of RA are capable of suppressing skin graft rejection [49], collagen induced arthritis [59] and allow the generation of mixed chimerism in transplant tolerance [63] suggesting that RA may also participate in the induction of other homing receptors.

In agreement with a role for RA in Treg induction, the administration of the pan-RAR antagonist LE540 in mice challenged with *Listeria monocytogenes* significantly reduces the number of mucosal Foxp3^+^ Tregs [64]. Similarly, in a model of experimental autoimmune uveitis, VAD mice exhibit a decreased frequency of intraocular Tregs [65]. Consistent with the effects produced by VAD, the administration of all-trans RA to normal mice leads to the expansion of Foxp3^+^ Tregs [66]. Moreover, *de novo* differentiation of Tregs from naive T cells is abrogated in VAD mice in a setting of oral tolerance [67], possibly due to a reduction in the trafficking of T cells to the intestine. It has been demonstrated that RA is crucial for T cell trafficking to the gut [27], and this migration is required for the expansion of Tregs during the induction of oral tolerance [68].

Despite converging evidence pointing towards a role for RA in the differentiation of Tregs *in vivo* and *in vitro*, some groups have reported that the percentage or absolute number of endogenous Tregs in the intestine are not altered in VAD mice [67,69]. Moreover, mice carrying a dominant-negative form of the RA receptor RARα in which RA signaling is impaired have a normal frequency and number of Tregs in the thymus and periphery [70]. Further studies are needed to fully understand the role of RA in the induction of extrathymic Tregs and its participation in oral tolerance.

## 5. Retinoic Acid in T Helper Cell Differentiation and Activation

Following activation, CD4^+^ T cells are able to differentiate into different T helper cell subsets that are classified according to their function, expression of lineage-specific master transcription factors, and secreted cytokines. Several T helper (Th) subsets have been described, which include Th1, Th2, Th17, T follicular helper (Tfh) and Tregs cells. CD4^+^ T cell differentiation into these different subsets is dependent on the cytokines present in the microenvironment during their activation [71]. 

Although overwhelming evidence supports the contribution of RA in tolerance via the negative regulation of the immune response through the induction or expansion of Tregs, recent evidence has shown that RA may also promote T cell activation and T helper cell responses during an ongoing immune response (Table A1 and Table A2). In this section, we will discuss recent evidence demonstrating a role for RA in T helper differentiation and activation.

### 5.1. Th17/Th1 Cell Differentiation

A wealth of evidence indicates that at pharmacological or high doses (10 nM and higher), RA has been shown to inhibit Th17 cell responses while inducing the generation of Tregs in murine models *in vitro* [46,61,64,72]. Moreover, the addition of high doses of RA has been shown to impair the differentiation of human Th17 and Th1 cells *in vitro* [73], suggesting that at pharmacological doses, RA is able to inhibit Th1 and Th17 cell differentiation in human CD4^+^ T cells. Importantly, other studies suggest that RA may have a dose-dependent effect over Th17 cell differentiation and lower or physiological concentrations of RA fail to inhibit Th17 cell differentiation [74,75]. Thus, although initially RA was considered detrimental for Th1 and Th17 responses, all these data suggests that RA inhibits Th1 and Th17 cell differentiation only at pharmacological doses by tilting the balance towards the expansion or generation of Tregs.

In contrast to the reports suggesting that RA inhibits Th1 and Th17 responses, some groups have reported that at low doses, RA favors Th1 and Th17 cell differentiation. Takahashi and collaborators have shown that at physiological doses (1nM), RA promotes Th17 cell differentiation *in vitro* [76]. In addition, the group of Belkaid demonstrated that Rarα^−/−^ T cells do not differentiate into Th1 or Th17 cells when cultured *in vitro* under Th1 or Th17 polarizing conditions [67], supporting a role of RA in the differentiation of Th1 and Th17 cells. Consistent with this evidence, several studies have reported that VAD mice show a significant impairment in Th1 and Th17 responses *in vivo* [67,69,75,77].

Other groups have suggested a role for RA in the induction of Th17 and Th1 responses during inflammation. It has been demonstrated that the addition of LE540, a RAR inhibitor, abrogates the *in vitro* Th17 cell differentiation induced by lamina propria DCs stimulated with flagellin [74]. In agreement, Rampal and collaborators have shown that retinoic-acid primed DCs can induce Th1 and Th17 cell differentiation *in vitro* [73]. Moreover, by studying the effects of IL-15 overproduction observed in celiac patients, DePaolo and collaborators found that RA synergizes with IL-15 to enhance IL-23 and IL-12 production by DCs, inducing Th1 cell polarization. Additionally, in the presence of IL-12 and IL-23, RA promotes Th1 and Th17 cell differentiation and inhibits Treg differentiation [78]. Furthermore, it has been demonstrated that RARα deficient naive T cells fails to acquire Th1 and Th17 phenotype in a model of transplantation [37] and that RARα deficient naive T cells fail to differentiate into Th1 cells in a model of intestinal inflammation [70]. Taken together, these observations have led to a model where RA may have a dual effect, favoring Treg-induced suppression of Th1 and Th17 responses in the steady state while inducing Th1 and Th17 cell mediated immunity during inflammation [22].

Additional effects of RA on other cells, such as the intestinal epithelium, may be important for the regulation of Th17 cells. It has been demonstrated that VAD mice present severe alterations in the growth and function of intestinal epithelial cells, including goblet cells, which enhances mucin production. This dysfunction alters the gut microbiota, resulting in the reduction in Th17 cell numbers in the small intestine [69].

### 5.2. Th2 Cell Differentiation

In the early 1990s, the group of Hayes demonstrated for the first time that VAD mice present an imbalance in Th1 and Th2 responses [79,80,81]. Using a parasite that induces Th2 responses in mice, they found that hypovitaminosis A contributes to IFN-γ overproduction, while dietary RA down-regulates Th1 responses and up-regulates Th2 responses *in vivo* [79]. In parallel, the group of Stephensen reached a similar conclusion when they reported that VAD impairs the salivary IgA response (associated with IL-4 production and Th2 responses) and at the same time enhances IgG2a serum levels (which is associated with IFN-γ production and Th1 responses) [82]. Moreover, a number of clinical studies on diet supplementation with vitamin A described a modest increase in the severity of respiratory infections in children suffering from pneumonia [83], suggesting that vitamin A may participate in the induction of Th2 responses while abrogating Th1 responses. In following studies, it was reported that high levels of dietary vitamin A enhances IgA and IL-10 production [84,85], while decreasing Th1 cell differentiation in mice [86], confirming that vitamin A promotes Th2 and inhibits Th1 responses *in vivo*.

To study the mechanisms that drive the above mentioned effect of RA on Th2 cell differentiation, Stephensen and collaborators performed *in vitro* studies where naive T cells were cultured in the presence of all-trans-RA, 9-cis-RA or an RXR agonist. Using this setting, they demonstrated that only 9-cis-RA and the RXR agonist were able to induce IL-4 production by T cells, demonstrating that Th2 differentiation occurs via stimulation of the RXR pathway [87]. In additional *in vitro* studies, Iwata and collaborators demonstrated that both all-trans-RA and 9-cis-RA enhances Th2 responses and that RAR but not RXR antagonists abrogates RA induction of Th2 responses [88].

The overwhelming evidence demonstrating an effect of vitamin A on Th2 cell differentiation (and thus, indirectly on antibody production) has led to the supplementation of vaccines with vitamin A with the aim to increase their efficacy. Despite this, results from human immunization programs have not shown a significant effect on vaccine efficacy upon vitamin A supplementation of vaccines [4].

Aside from its effect on the Th2 adaptive immune response, recent evidence supports a role of vitamin A on the regulation of innate immune responses. The Belkaid group has shown that in contrast to the common belief that VAD is associated with global immunosuppression, the immune system is able to adapt and induce a type 2 response mediated by innate lymphoid cells (ILCs). In this study, they show that although Th2 and other T helper responses are abrogated in VAD mice, type 2 innate lymphoid cells (ILC2) are expanded, providing resistance to nematode infection in mice. Type 2 responses elicited by ILC2 cells are particularly essential for eradicating worm and parasite infections, which are prevalent in regions associated with high levels of malnutrition [77].

### 5.3. T Cell Activation

Although previous evidence has shown that RA is mainly produced by cells from the gut [27,35,36], some reports have shown that RA may also be produced at other sites during an ongoing immune response [36,37,89,90]. Using RA signaling reporter mice (RARE-luciferase reporter mice), Pino-Lagos and collaborators showed that RA signaling occurs constitutively in areas of the small and large intestine. Importantly, the intradermal administration of activating stimuli such as LPS elicited local and transient RA signaling in the site of inflammation [37]. This finding supports a role for RA in T cell activation during an inflammatory immune response. Similarly, Hall and collaborators have demonstrated that RA-RARα signaling is required for early T cell activation as RARα-deficient T cells proliferate less efficiently than their wild-type counterparts upon polyclonal activation. They further demonstrated that RARα mediates events following T cell receptor activation since RARα-deficient T cells present decreased levels of PLCγ and ERK phosphorylation, Ca^2+^ mobilization and mTOR/AKT activation upon T cell receptor stimulation [67]. These results strongly argue in favor of RA as an essential metabolite in the development of an appropriate immune response.

## 6. Role of Retinoic Acid in Thymus and Lymphoid Organogenesis

Thymocytes differentiate into mature T cells in the thymus, where they generate and express a unique T cell receptor. T cells expressing a potentially autoreactive T cell receptor are eliminated by apoptosis in a phenomenon known as negative selection [91]. Since the initial report of Wolbach in the early 20th century demonstrating that VAD is associated with thymic atrophy [92], several potential functions for RA in thymocyte development have been proposed. Kiss and collaborators have shown that RALDH1 is expressed by cortical and medullary thymic epithelial cells, and accordingly, all-trans-RA was detected in the murine postnatal thymus [93]. Iwata and collaborators have shown that RA inhibits TCR-mediated activation-induced thymocyte cell death *in vitro*, suggesting that this metabolite might affect T cell differentiation and negative selection in the thymus [94]. Following this report, a study using fetal thymic organ cultures showed that RA increases the proportion of mature CD4^+^ T cells that contain autoreactive T cells [95]. Using RARα-selective agonists, it was demonstrated that RARα signaling is involved in the prevention of activation-induced cell death in thymocytes [96]. On the other hand, the same group reported that RA can induce thymocyte apoptosis when these cells are not activated, and this effect is mediated through RARγ signaling [97]. Moreover, the treatment of mice with a RARγ agonist induces rapid thymic involution [97]. These observations raise the possibility that RA may have an effect on negative selection in the thymus thus shaping the T cell repertoire in adults.

Lymphoid tissue inducer cells (LTi) constitute a subset within ILCs closely related to type 3 ILCs (ILC3s). LTi cells are essential for lymphoid organogenesis during embryogenesis, and they are also considered important regulators of lymphoid tissue architecture after birth [98,99,100]. Similar to Th17 cells, LTi cells are controlled by the RORγt transcription factor, and thus, it has been reported that RORγt-deficient mice do not have LTi cells and have important alterations in secondary lymph node development [101]. Recent evidence has noted a relevant role of maternal vitamin A intake in the immune fitness of the offspring. Van de Pavert recently reported a cross-talk between the developing neuronal system and hematopoietic stromal cells since the initiation of lymph node development is controlled by the RA-mediated expression of CXCL13 by adjacent neurons, promoting the migration of LTi cells [102]. In a following report, the same group demonstrated that local LTi cell differentiation is controlled by maternal vitamin A intake and fetal RA signaling. In this latter study, the authors demonstrate that maternal retinoids control LTi differentiation within the developing lymph nodes. Moreover, impaired RA signaling results in reduced fetal inguinal and brachial lymph node size and a reduced numbers of Peyer’s patches [102]. This report reached the significant conclusion that the efficiency of adaptive immune responses against infections may be somehow pre-established during embryogenesis through vitamin A ingestion and the nutritional status of the mother.

Recently, Zhang *et al.* established that the gut microbiota, in particular gut fungi, is required for secondary lymphoid organ development through the induction of the migration of CD103^+^ DCs into the gut from the periphery. These CD103^+^ DCs produce RA and signal to initiate an increase in lymph node cellularity and volume. Interestingly, they report that VAD in 5 week-old mice severely disrupts the peripheral lymph node architecture, establishing not only that gut-associated lymph nodes may be regulated by vitamin A, as shown by van de Pavert, but also that vitamin A may be involved in the postnatal regulation of peripheral lymph node structure [103].

## 7. Concluding Remarks

An initial association between vitamin A supplementation and reduced childhood mortality due to measles and diarrhea led to the assumption that vitamin A was crucial in the fitness of the immune system. This finding sparked curiosity about the mechanisms through which vitamin A could modulate the immune response. Today, it is recognized that vitamin A, through its metabolite retinoic acid, is able to induce gut homing receptors on T and B cells, allowing the trafficking of these cells to the intestine (and probably also to the inflamed tissues) to perform their effector functions and maintain an appropriate gastrointestinal balance between immunity and tolerance. Moreover, exposure to vitamin A in the womb is an important determinant for the development of secondary lymphoid organs during embryogenesis and after birth through induction of lymphoid tissue-inducer cells. In addition, vitamin A has shown to be essential for T cell activation and differentiation into different T helper subsets, such as Th1, Th2 and Th17 cells. On the other hand, *in vitro* studies have shown that RA is able to induce regulatory T cells, however the role of vitamin A in promoting oral tolerance has not yet been fully established. A far more complex picture has emerged for the role of retinoic acid in gut immunity since there is contradictory evidence for several of aspects of T cell differentiation. Such discrepancies could be explained by the fact that RA can mediate different effects depending on the dose used in different experimental settings and the context in which an immune response is occurring. Accordingly, it has been proposed that RA may have a dual effect, modulating regulatory T cell differentiation in the steady state while promoting T cell activation and Th1 and Th17 cell responses during an ongoing immune response. In addition, results in VAD mice should be interpreted with caution; taking into consideration that VAD mice present important alterations in intestinal epithelial cells, which affects the resident microbiota. The gut microbiota has an important effect on the differentiation of T helper subsets and Tregs in the gut. Therefore, the effects observed in VAD mice could be the consequence of an altered gut microbiota rather than a direct effect of RA on T cells. Although the development of better models and experimental techniques has allowed significant progress in the discovery of the mechanisms through which vitamin A broadly impacts the immune response, further studies are needed to fully unravel the role of vitamin A in T cell-mediated immunity.

## Appendix

**Table A1:** Effects of RA signaling on CD4^+^ T cell differentiation *in vitro*.

T Cell Subset	RA Effect	Experimental Setting	Model Organism [Refs]
Treg	Induces Treg differentiation	Cultures in the presence of RA (0.05 nM–10 μM), RAR agonist (AM580) or antagonists (Ro41-5253, LE540, LE135)	Mice [35,45,46,47,48,49,50,58,61,72] Human [47,52,54]
Treg	Enhances Treg stability and function	Adoptive transfer of Treg generated in the presence of RA in mice models of inflammation (immunization, transplant, intestinal inflammation, GvHD)	Mice [49,50,58] Human [52,54]
Th17	Induces Th17 differentiation	Cultures in the presence of physiological doses of RA (1 nM) or RAR antagonist (LE540). Cultures of RARα^−/−^ CD4^+^ T cells	Mice [67,74,76]
Th17	Reduces Th17 differentiation	Cultures in the presence of pharmacological doses of RA (≥10 nM), RAR agonist (TTNBP) or antagonist (LE135)	Mice [46,47,64,61,69,72,74,75,76] Human [73]
Th1	Induces Th1 differentiation	Cultures of RAR deficient CD4^+^ T cells	Mice [67,70]
Th1	Enhances Th1 stability	Ablation of RA signaling in Th1 committed cells	Mice [70]
Th1	Reduces Th1 differentiation	Cultures in the presence of pharmacological doses of RA (≥10 nM), RXR agonists (HX600, TZ335, PA024), RAR agonists (Am80, Tp80), RXR antagonist (PA452) or RAR antagonists (LE135, LE540)	Mice [72,74,85,87] Human [73]
Th2	Induces Th2 differentiation	Cultures in the presence of 9-cis RA (10 nM–1 μM), RXR agonists (AGN194204, HX600, TZ335, PA024), RAR agonists (Am80, Tp80), RXR antagonist (PA452) or RAR antagonists (LE135, LE540)	Mice [85,87,88]
Th2	Reduces Th2 differentiation	Cultures in the presence of pharmacological doses of RA (≥100 nM)	Mice [72]

**Table A2:** Effects of RA signaling on CD4^+^ T cell differentiation *in vivo*.

T Cell Subset	RA Effect	Experimental Setting [Refs]
Treg	Induces Treg differentiation	*In vivo* administration RAR antagonist (LE540) in in *L. monocytogenes* infected mice [64] VAD mice after induction of experimental autoimmune uveitis [65] *In vivo* administration of RA in mice model of diabetes [66] VAD mice after oral tolerance induction [67]
Treg	No effect on frequencies or numbers of endogenous Treg	Frequencies and/or absolute numbers of endogenous Treg in VAD mice [67,69] and RARα^−/−^ mice [67]
Th17	Reduces Th17 differentiation	Frequencies of Th17 cells in small intestine lamina propria after oral administration of RA in *L. monocytogenes* infected mice [64] Endogenous response to *L. monocytogenes* in CD4 conditional RARα-deficient mice [70] CD4 conditional RARα-deficient mice in spleen at steady state [70] *In vivo* differentiation of RARα deficient CD4^+^ T cells in mice model of intestinal inflammation [70] Frequencies of Th17 cells in spleen after intraperitoneal administration of RA in experimental autoimmune encephalomyelitis mice [61]
Th17	Induces Th17 differentiation	Frequencies of Th17 cells in small intestine [69,75] and Peyer’s patches in VAD mice in steady state [75] Frequencies of Th17 cells in small intestine lamina propria after immunization (OVA + LTR129G) in VAD mice [67] Frequencies of Th17 cells in large intestine lamina propria after infection with *C. rodentium* in VAD or RAR antagonist BMS493-treated mice [77] Frequencies of Th17 cells in VAD mice after infection with *T. gondii* [67] *In vivo* differentiation of RARα deficient CD4^+^ T cells in mice model of transplantation [37]
Th1	Induces Th1 differentiation	CD4 conditional RARα-deficient mice in spleen at steady state [70] Endogenous response to *L. monocytogenes* in CD4 conditional RARα-deficient mice [70] *In vivo* differentiation of RARα deficient CD4^+^ T cells in mice model of intestinal inflammation [70] *In vivo* differentiation of RARα deficient CD4^+^ T cells in mice model of transplantation [37] *In vivo* differentiation of CD4^+^ T cells after immunization with cognate antigen in VAD mice [86]
Th1	Impairs Th1 responses	Influenza-specific IgG and IFN-γ production after intranasal inoculation with influenza virus in VAD mice [82] or mice fed with a vitamin A-rich diet [84] Antigen-specific IFN-γ production in mesenteric lymph node and spleen cells after infection with *T. spiralis* in VAD mice [79,81]
Th2	Induces Th2 differentiation	Influenza-specific IgA and IL-10 production after intranasal inoculation with influenza virus in VAD mice [82] or mice fed with a vitamin A-rich diet [84] Antigen-specific IL-4, IL-5 production in mesenteric lymph node cells after infection with *T. spiralis* in VAD mice [79,81] *In vivo* differentiation of CD4^+^ T cells after immunization with cognate antigen in VAD mice [86]
